# Large-scale analysis of protein expression changes in human keratinocytes immortalized by human papilloma virus type 16 E6 and E7 oncogenes

**DOI:** 10.1186/1477-5956-7-29

**Published:** 2009-08-23

**Authors:** Mark A Merkley, Ellen Hildebrandt, Robert H Podolsky, Hilal Arnouk, Daron G Ferris, William S Dynan, Hubert Stöppler

**Affiliations:** 1 Institute of Molecular Medicine and Genetics, Medical College of Georgia, Augusta, GA, USA; 2 Center for Biotechnology and Genomic Medicine, Medical College of Georgia, Augusta, GA, USA; 3 Department of Obstetrics and Gynecology, Medical College of Georgia, Augusta, GA, USA; 4 Department of Family Medicine, Medical College of Georgia, Augusta, GA, USA; 5 Department of Microbiology and Immunology, Louisiana State University Health Sciences Center, Shreveport, Louisiana 71130, USA; 6 Division of Hematology/Oncology, Department of Medicine, University of Alabama at Birmingham, Birmingham, Alabama 35294, USA

## Abstract

**Background:**

Infection with high-risk type human papilloma viruses (HPVs) is associated with cervical carcinomas and with a subset of head and neck squamous cell carcinomas. Viral E6 and E7 oncogenes cooperate to achieve cell immortalization by a mechanism that is not yet fully understood. Here, human keratinocytes were immortalized by long-term expression of HPV type 16 E6 or E7 oncoproteins, or both. Proteomic profiling was used to compare expression levels for 741 discrete protein features.

**Results:**

Six replicate measurements were performed for each group using two-dimensional difference gel electrophoresis (2D-DIGE). The median within-group coefficient of variation was 19–21%. Significance of between-group differences was tested based on Significance Analysis of Microarray and fold change. Expression of 170 (23%) of the protein features changed significantly in immortalized cells compared to primary keratinocytes. Most of these changes were qualitatively similar in cells immortalized by E6, E7, or E6/7 expression, indicating convergence on a common phenotype, but fifteen proteins (~2%) were outliers in this regulatory pattern. Ten demonstrated opposite regulation in E6- and E7-expressing cells, including the cell cycle regulator p16^INK4a^; the carbohydrate binding protein Galectin-7; two differentially migrating forms of the intermediate filament protein Cytokeratin-7; HSPA1A (Hsp70-1); and five unidentified proteins. Five others had a pattern of expression that suggested cooperativity between the co-expressed oncoproteins. Two of these were identified as forms of the small heat shock protein HSPB1 (Hsp27).

**Conclusion:**

This large-scale analysis provides a framework for understanding the cooperation between E6 and E7 oncoproteins in HPV-driven carcinogenesis.

## Background

The oral cavity, oropharynx, larynx, esophagus, and ano-genital orifices are lined with stratified squamous nonkeratinized epithelium, which forms the barrier between the underlying tissue and the environment. The proliferative nature of this epithelium, together with its potential exposure to environmental insults such as oncogenic viruses makes it susceptible to carcinogenesis. Indeed, carcinomas of stratified squamous nonkeratinized epithelium are among the most common and deadly cancers worldwide. Cervical squamous cell cancer is the second leading cause of death among women and is responsible for loss of 3.3 million life-years annually. Although head and neck squamous cell cancer is a more heterogeneous disease, it is the sixth most commonly diagnosed malignancy worldwide and also imposes a significant global health burden.

Infection with high-risk subtype mucosatropic human papillomavirus (HPV) is associated with 99.7% of cervical cancers [1,2] and for a subset of head and neck squamous cell carcinomas and anal squamous cell carcinomas [3-8]. Expression of the HPV E6 and E7 oncoproteins promotes neoplastic transformation by altering expression or interfering with the function of proteins involved in cell proliferation and apoptosis (reviewed in [9,10]). E6 expression influences the stability or function of proteins including TP53, hScrib, hDlg, MUPP1, p300, NF-κb, and IRF-3 [11-13]. Many of the effects of E6 are attributable to its interaction with E6-associated protein (E6AP), an E3 ubiquitin ligase, although some effects are E6AP-independent [14-16]. E7 binds to the retinoblastoma protein (Rb) and disrupts the Rb/E2F/HDAC complex. This abolishes the transcriptional trans-repressor functions of the complex and leads, via E2F release, to the induction of the transcriptional trans-activation function of E2F (reviewed in [17]). Additionally, E7 binds directly to cyclin A- and E-dependent kinase complexes, and E7-dependent inhibition of the cyclin-dependent kinase inhibitors p21 and p27 has been demonstrated [17-19]. Both E6 and E7 have been shown to play a role in the suppression of the immune response to infection [20,21].

Expression of either high-risk HPV E6 or E7 in human keratinocytes extends the period of growth prior to senescence well beyond normal. Combined expression of E6 and E7, however, is more efficacious than their individual expression in promoting cellular immortalization [10,15,22-25]. The two viral oncogenes target different cellular regulatory pathways, and their combined expression induces cell proliferation and simultaneously suppresses the apoptotic response associated with oncogene-induced unscheduled cell proliferation.

We report here the results of a large-scale analysis to quantify the extent to which proteomic profiles differ from each other in cells that have been immortalized by the expression of E6 or E7 individually and in combination. We used an *in vitro *model consisting of primary human foreskin keratinocytes (HFKs) immortalized by transduction with HPV oncogenes [26]. The methodology used for our study was 2D-differential gel electrophoresis (2D-DIGE), which involves co-electrophoresis of experimental samples with a differentially labeled internal standard [27]. This technique has been widely applied previously for clinical proteomics, providing a basis for comparison between results in the *in vitro *model and clinical studies. Proteomic methods have been used previously to characterize E6- and E7-associated proteins. Two studies identified proteins modulated by transfection of E7 [28,29] and one study identified proteins modulated by transfection of E6 [30]. However, these were based on expression of viral oncogenes E6 and E7 individually into established cancer cell lines. The earlier studies did not include the comparison to primary cells and to cells expressing both oncogenes simultaneously that provide the underlying analytical framework in the present study.

We determined that 170 out of 741 spots (23%) were significantly different in abundance in immortalized cells versus HFKs. The overwhelming majority of these showed qualitatively similar changes regardless of which oncogene drove the immortalization. We assume that the vast majority of these alterations are not directly associated with viral oncogene expression, but that they are rather a consequence of cell immortalization. The most interesting features of the data set may be the small number of outliers that did not follow this general trend, including ten protein features oppositely regulated in E6- vs. E7-transduced cell populations, compared to HFKs, and five that showed significantly higher expression in the E6/7-transduced population than was expected, based on results in cells transduced with E6 or E7 alone.

## Results

### 2D-DIGE analysis

The method of generating HFK-derived cell populations expressing HPV 16 E6, E7, or both E6 and E7 (E6/7) oncoproteins has previously been described [26]. Continuous cultivation leads to the establishment of immortalized keratinocyte cultures expressing the HPV oncogenes and derived from a genetically identical host background [26]. Cell lysates of the immortalized HPV oncogene-expressing cultures and a primary, non-HPV oncogene-expressing HFK culture were harvested for proteome characterization.

**Figure 1:**
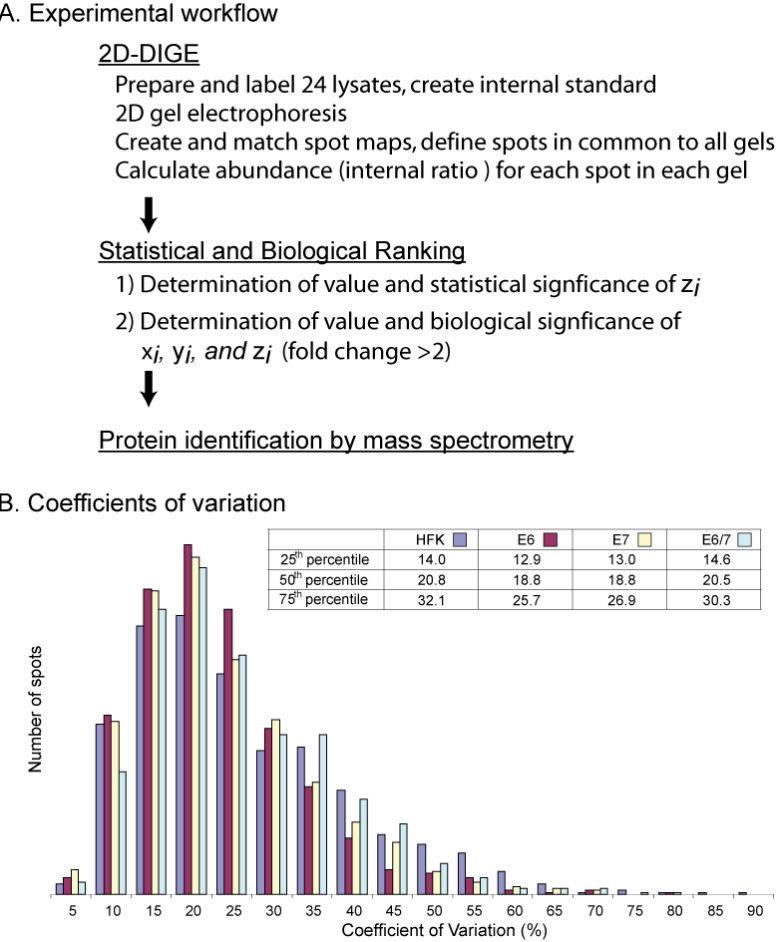
**Workflow and quality control metrics**. A. Experimental workflow. B. Histogram showing coefficients of variation. Colors distinguish HFK, E6, E7, and E6/7 groups as indicated in the key.

Figure 1 illustrates the experimental workflow and quality control metrics for the study. There were 24 analytical gels in the experiment, representing six replicates of each of the four experimental groups: non-immortalized HFKs, E6-transduced keratinocytes, E7-transduced keratinocytes, and E6/7-transduced keratinocytes. Each gel contained 1 μg of cell lysate from an experimental sample labeled with Cy5 and 1 μg of internal standard labeled with Cy3 dye. The internal standard consisted of an equal mixture of cell lysates from all 4 experimental groups (for further details on experimental design see Methods and Figure 1A). Saturation cysteine labeling (as opposed to minimal labeling at lysine residues) was chosen so that spot mobility would be as similar as possible to a prior clinical study [31]. Following two-dimensional electrophoresis, DeCyder software was used to create a spot map for each gel. The presence of the invariant internal standard facilitated matching spots across the gel set. An average of 1812 spots per gel matched to the master spot map, and 741 spots were common to all gels. For each of these 741 spots, we calculated relative abundance as described in the Methods section. For each spot, we then determined the mean increments in expression in E6-transduced cultures, E7-transduced cultures, and E6/7 transduced cultures, relative to HFKs. We expressed these parameters on a log_2_-transformed scale and designated them as x_*i*_, y_*i*_, and w_*i*_, respectively, where *i *is the spot number (i.e., a unit increment in x_*i*_, y_*i*_, and w_*i *_represents a two-fold change in relative abundance). We defined a fourth parameter, z_*i*_, as the difference between the actual increment in expression in E6/7-transduced cells and the predicted increment based on the sum of x_*i *_and y_*i*_. Based on this definition, we shall refer to z_*i *_as an "E6/7 interaction" parameter. The derivation of these parameters from the experimental data is explained in more detail in Additional file 1.

To evaluate reproducibility of the abundance measurements, we determined within-group coefficients of variation (CVs) (Figure 1B). The median CV for each group ranged from 18.8% to 20.8%, indicating that within-group variation was small, relative to the anticipated between-group differences.

### Identification of proteins of interest

To identify the most interesting features in the data set, we characterized proteins according to statistical and biological significance as detailed in Additional file 1. Briefly, Significance Analysis of Microarray (SAM) was first used to classify the spots according to whether the E6/7 interaction parameter z_*i *_was significant (z_*i *_≈ 0 versus z_*i *_≠ 0). For spots where z_*i *_≈ 0, we were able to use statistically powerful group comparisons and SAM analysis to evaluate x_*i *_and y_*i*_. We used a false discovery rate (FDR) < 5% as a threshold for statistical significance, and |x_*i*_| or |y_*i*_| > 1 (corresponding to a minimum 2-fold change associated with E6 or E7 expression) as a threshold of biological significance. For spots where statistical significance of z_*i *_was established by the initial SAM analysis (E6/7-associated increment in expression significantly more or less than predicted by the sum of the E6 and E7-associated increments, x_*i *_and y_*i*_), a value of |x_*i*_| or |y_*i*_| > 1 was used as a further test of biological significance. Based on these criteria, 170/741 spots (23%) were evaluated as significant. These could be classified in eight combinatorial groups based on the algebraic sign of x_*i*_, y_*i*_, and z_*i *_(Table 1).

**Table 1:** Significant spots categorized by expression pattern

**Trend**	**x**_*i*_	**y**_*i*_	**z**_*i*_	**n (spots)**	**n (significant)**	**Examples**
Up-regulated in oncogene-expressing cells	>0	>0	<0	325	57	Oncoprotein DJ-1, ezrin, multiple HSPs, metabolic, and regulatory proteins
	
	>0	>0	>0	31	5	HSPB1 (2 forms), peroxiredoxin-3

Mixed regulation in oncogene-expressing cells	>0	<0	<0	21	4	HSPA1
	
	>0	<0	>0	38	0	(none)
	
	<0	>0	>0	49	3	p16^ink4a^, keratin 7 (2 forms)
	
	<0	>0	<0	69	3	Galectin-7

Down-regulated in oncogene-expressing cells	<0	<0	<0	10	1	(none)
	
	<0	<0	>0	198	97	Serpin B5, annexin A2, 14-3-3σ, lamin A/C multiple cytokeratins

Total				741	170	

All 741 protein spots were classified according to the algebraic sign of x_*i*_, y_*i*_, and z_*i*_. For each of eight possible permutations, the table provides the total number of spots and the number of spots evaluated as potentially significant using statistical and biological criteria specified in Additional file 1. The table also provides examples of identified proteins in each category.

**Figure 2:**
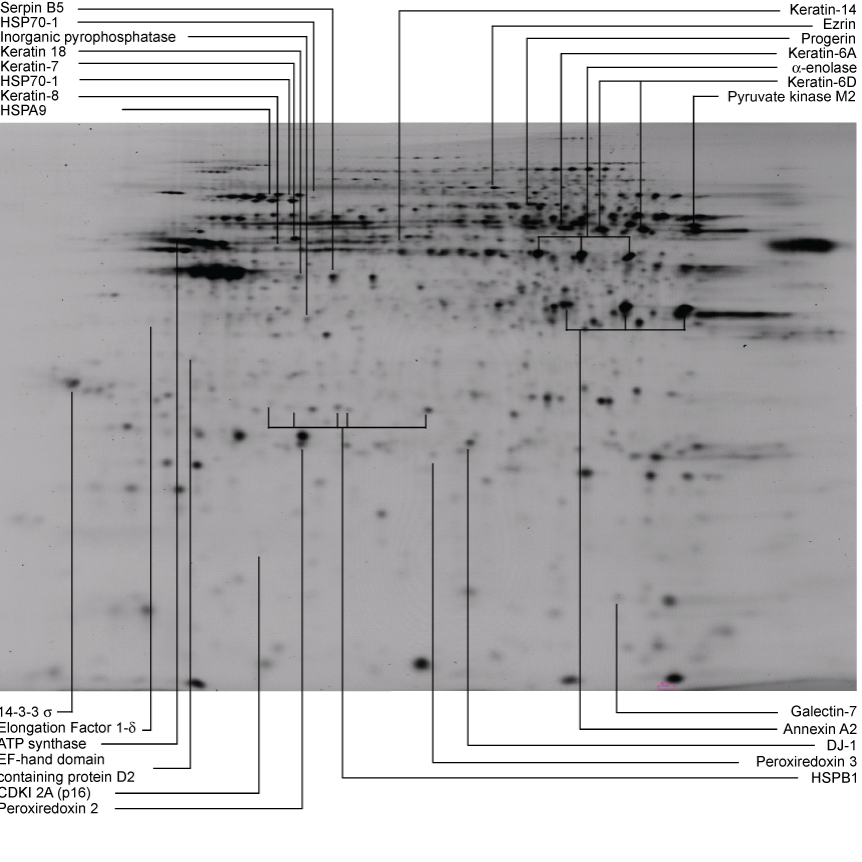
**Identification of protein spots on 2D gel**. Image depicts Cy3 (internal standard) channel for a representative gel. Proteins from each sample group and the internal standard were separated in two dimensions. Horizontal dimension is isoelectric focusing (pH3-10, acidic end to left). Vertical dimension is 12.5% SDS-PAGE. Indicated spots were identified with high confidence and met criteria for statistical and biological significance. Some proteins were identified more than once because charge isoforms were present.

We selected 65 significant spots for mass spectrometry analysis. We ran a separate preparative gel, matched it to the master analytical gel, and picked and identified spots as described in Materials and Methods. We identified 42 spots (65%) with a Mascot score > 113 (confidence level of identification 100%), 13 (20%) with a Mascot score of 65–112 (confidence level of identification > 95%), and failed to identify 10 spots (15%). Figure 2 shows a subset of the identified proteins projected on a representative 2D gel image. There were several instances where nearby spots were identified as the same protein, probably reflecting charge modification. All identified proteins migrated within a range consistent with expected mass and pI values. Identified proteins are listed in Table 2, with further details of the identification listed in Additional file 1, Table S1.

**Table 2:** Spots identified by mass spectrometry

Group	spot number	Protein name	Gene name	UniProt Accession Number	DC-1	DC-2	GC-C	DC-3
					2^x*i*^	2^y*i*^	2^z*i*^	2^w*i*^
Down-regulated 2^x*i *^< 1, 2^y*i *^< 1	572	Keratin, type II cytoskeletal 6C	KRT6C	P48668	0.32	0.19	4.47	0.27
	
	560	Keratin, type II cytoskeletal 6C	KRT6C	P48668	0.35	0.21	4.13	0.3
	
	545	Pyruvate kinase isozyme M2	PKM2	P14618	0.37	0.14	7.14	0.36
	
	1110	Annexin A2^b^	ANXA2	Q8TBV2	0.38	0.66	1.36	0.34
	
	775	ATP synthase subunit b, mitochondrial	ATP5B	P06576	0.38	0.13	7.79	0.39
	
	2967	Keratin, type II cytoskeletal 6A	KRT6A	P02538	0.39	0.27	3.12	0.33
	
	672	Keratin, type I cytoskeletal 14	KRT14	P02533	0.4	0.27	3.09	0.33
	
	1111	Annexin A2^b ^(lipocortin)	ANXA2	Q8TBV2	0.41	0.68	1.39	0.39
	
	439	Progerin (Lamin A/C)	LMNA	Q6UYC3	0.41	0.54	2.77	0.62
	
	534	Keratin, type II cytoskeletal 6A	KRT6A	P02538	0.43	0.22	4.5	0.42
	
	460	Pyruvate kinase isozyme M2	PKM2	P14618	0.46	0.58	2.58	0.69
	
	739	Keratin, type II cytoskeletal 8	KRT8	P05787	0.5	0.3	3.49	0.53
	
	1586	14-3-3 protein σ (stratifin)	SFN	P31947	0.53	0.49	1.82	0.47
	
	903	Serpin B5 (maspin)	PI5	P36952	0.7	0.45	1.6	0.51
	
	915	Keratin, type I cytoskeletal 18	KRT18	P05783	0.76	0.48	1.75	0.65

Mixed regulation 2^x*i *^< 1, 2^y*i *^> 1, OR 2^x*i *^> 1, 2^y*i *^< 1	2402	Cyclin-dependent kinase inhibitor 2A, isoforms 1/2/3 (p16)	CDKN2A	P42771	0.34	4.83	3.46	5.77
	
	645	Keratin, type II cytoskeletal 7^c^	KRT7	P08729	0.37	2.32	1.53	1.31
	
	646	Keratin, type II cytoskeletal 7	KRT7	P08729	0.3	1.6	2.06	0.97
	
	2597	Galectin-7	LGALS7	P47929	0.37	2.51	0.25	0.23
	
	366	HSPA1A	HSPA1A	P08107	3.01	0.86	0.71	1.86

Up-regulated 2^x*i *^> 1, 2^y*i *^> 1	1451	EF-hand domain containing protein D2	EFHD2	Q96C19	1.17	2.06	0.59	1.42
	
	266	Ezrin (villin-2)	VIL2	P15311	1.5	2.55	0.45	1.7
	
	1685	HSPB1 (Hsp27)	HSPB1	P04792	2	1.14	0.44	1.01
	
	2983	Elongation factor 1-δ	EEF1D	P29692	2.08	1.84	0.68	2.58
	
	1186	Inorganic pyrophosphatase	PPA1	Q15181	2.26	1.4	0.58	1.82
	
	1721	Keratin, type I cytoskeletal 10	KRT10	P13645	2.34	2.58	0.26	1.56
	
	1694	HSPB1	HSPB1	P04792	2.48	1.08	0.47	1.25
	
	777	α-enolase	ENO1	P06733	2.52	1.25	0.41	1.3
	
	781	α-enolase	ENO1	P06733	2.57	1.27	0.34	1.12
	
	778	α-enolase	ENO1	P06733	2.6	1.28	0.34	1.15
	
	1849	Protein DJ-1	PARK7	Q99497	2.79	1.43	0.31	1.22
	
	382	HSPA9 (mortalin, GRP 75)	HSPA9	P38646	2.92	1.29	0.31	1.17
	
	766	α-enolase	ENO1	P06733	3.27	1.49	0.26	1.25
	
	1859	Protein DJ-1	PARK7	Q99497	3.93	1.58	0.23	1.45
	
	377	HSPA1A (Hsp70-1)	HSPA1A	P08107	4.08	1.65	0.21	1.4
	
	1678	HSPB1 (Hsp27)	HSPB1	P04792	6.95	1.13	0.24	1.86
	
	1686	HSPB1 (Hsp27)	HSPB1	P04792	1.04	1.45	1.91	2.88
	
	1839	Thioredoxin-dependent peroxide reductase, mitochondrial^c^	PRDX3	P30048	1.66	1.54	1.01	2.58
	
	1663	HSPB1^c ^(Hsp27)	HSPB1	P04792	4.04	1.59	2.8	17.97

DC-1, DC-2, and DC-3 stand for direct comparisons 1, 2, and 3. GC-C stands for grouped comparison C. All proteins met criteria for significance. ^b^Significant in group comparison A. ^c ^Significant in both group comparisons A and B. Proteins are grouped by the direction of expression for the viral oncogene-transduced cultures as compared to non-viral oncogene-expressing primary HFKs. Names and accession numbers are in the UniProt format. Note that by definition, z_*i *_= w_*i*_. - (x_*i *_+ y_*i*_). For this table, the x_*i*_, y_*i*_, z_*i*_, and w_*i *_values have been exponentiated (i.e., converted to fold change) to facilitate interpretation. Proteins with values < 0.5 and > 2.0 represent a 2-fold change (absolute value of x_*i *_or y_*i *_> 1). Group symbols correlate with the symbols used in Figure 4.

### Correlation of 2D-DIGE analysis and immunoblotting results

**Figure 3:**
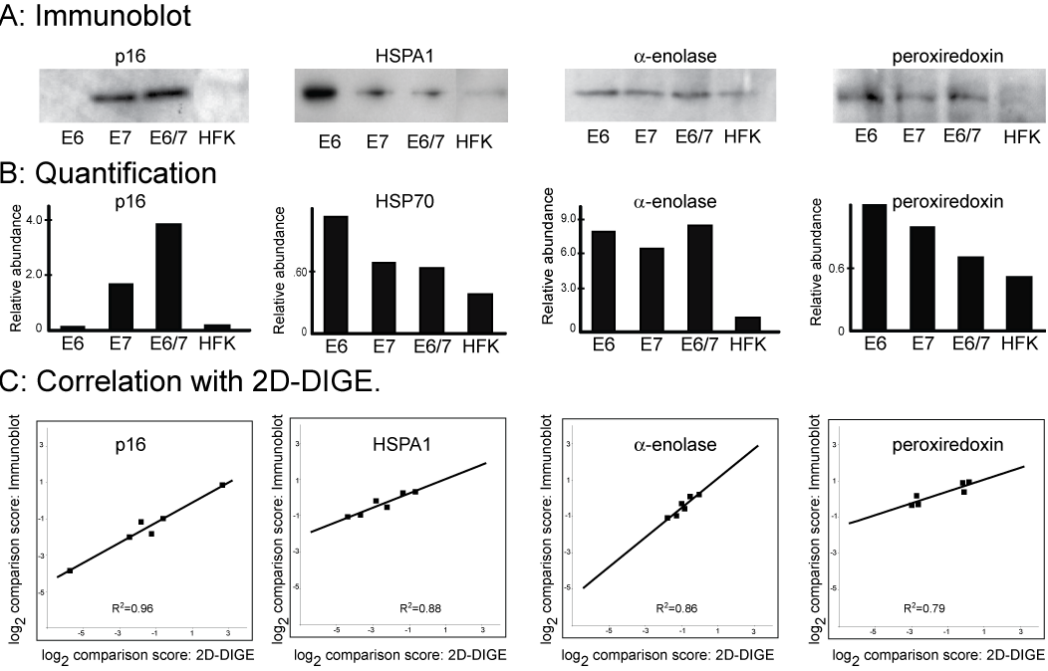
**Correlation of immunoblot analysis and 2D-DIGE**. A. Immunoblotting was performed as described in the Methods section using lysates from the same cells as for 2D-DIGE. For HSPA1, 5 μg of protein extract was analyzed by 8% SDS-PAGE. For all others, 30 μg extract was analyzed by 14% SDS-PAGE. Immunoblots were developed using a fluorogenic dye (ECF, GE Healthcare). B. Quantification of the immunoblots in Panel A by Kodak 1D 3.5 imaging software. C. Correlation between 2D-DIGE and immunoblot results. Six intergroup comparisons were made. Three were individual comparisons: E6 v. HFK, E7 v. HFK, and E6/7 v. HFK. Three were grouped comparisons: sum of HFK and E6 v. sum of E7 and E6/7, sum of HFK and sum of E7 v. E6 and E6/7, and sum of HFK and E6/7 v. sum of E6 and E7.

We selected four identified proteins for confirmatory immunoblot analysis (Figure 3). Proteins were chosen based on the availability of high quality antibodies and on a preliminary analysis of the 2D-DIGE data. We carried out individual and grouped comparisons as described in the legend to Figure 3, using values obtained by 2D-DIGE or immunoblotting. The scatter plot (panel C) indicates the relationship obtained by using each data set. Values were plotted on a log-transformed scale such that a unit increment on each axis corresponds to a 2-fold change in relative abundance. For each protein, a strong correlation (r^2 ^> 0.8) was seen. We noted that, although values were highly correlated, the slopes of the best-fit lines were uniformly < 1 (i.e., the magnitude of differences measured by immunoblotting are less than those measured by 2D-DIGE). We suspect that the quantitative discrepancy with the 2D-DIGE and immunoblotting results arises because of the limited dynamic range of the film-based method for detecting the ECL signal (Methods).

### Analysis of expression patterns

**Figure 4:**
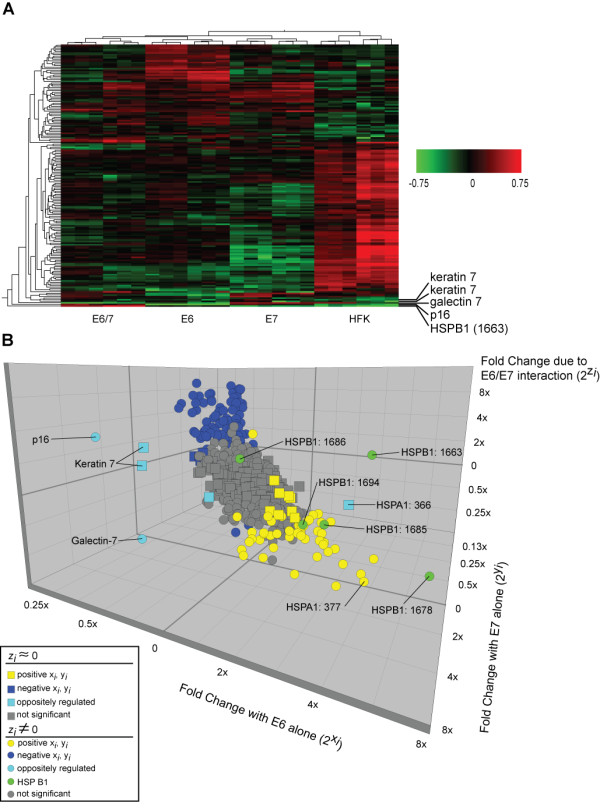
**Proteomic patterns. **Three-dimensional scatter plot of entire proteomic data set. **A. Results of cluster analysis represented as heat map.** Proteomic features that changed significantly in immortalized versus HFK populations were analyzed by unsupervised cluster analysis. Experimental samples are clustered on the horizontal axis and protein spots on the vertical axis. Legend indicates fold change on log_10 _scale. **B. Three-dimensional scatter plot of entire proteomic data set.** Axes represent fold change in expression due to E6 alone (2^x*i*^), E7 alone (2^y*i*^), and the E6/7 interaction (2^z*i*^). Grey spots did not reach criteria for significance. Other spots denote proteins that were significantly up-regulated (yellow), down-regulated (blue), or showed a mixed pattern of regulation (turquoise) in E6- and E7-transduced populations (i.e., x_*i *_> 0 and y_*i *_< 0 or x_*i *_< 0 and y_*i *_> 0). Different forms of HSPB1 were assigned a common color (green) to aid in visualization. Charge isoforms of HSPA1 and HSPA1 are labeled according to spot number in the master spot map. Shape of symbols denotes significance of z_*i *_(squares, not significant; circles, significant). For clarity, labels have been omitted for identified proteins in the large clusters of spots that were similarly regulated.

To help visualize large-scale patterns in the data, we generated a heatmap by unsupervised clustering using the 170 protein features that met criteria for significance (Figure 4A). The primary HFKs form a clear outgroup, whereas the immortalized populations can be seen to have converged on a broadly similar phenotype. The six replicates in each experimental group (E6, E7, and E6/7) cluster together, and within each experimental group the technical replicates (collected at the same population doubling level) cluster more closely than the biological replicates (clustered at a subsequent population doubling).

To more clearly distinguish outliers in the overall proteomic pattern, we plotted x_*i*_, y_*i*_, and z_*i *_parameters associated with all 741 spots in the study as a three-dimensional graph (Figure 4B). Most spots (>97%) fall into a "main sequence" – a cluster with near-continuous distribution of x_*i*_, y_*i*_, and z_*i *_values. At the center of the cluster (grey squares) are spots with expression changes that were not significant by any criterion. Surrounding this core are spots where z_*i *_≠ 0 but where effects of E6 and E7 transduction were too weak to meet the threshold of biological significance (grey circles). Neither of these groups was characterized further.

In front and below the central grey spots are 57 spots (yellow) that were evaluated as significant and share the common property that x_*i *_> 0, y_*i *_> 0. That is, expression was up-regulated in both E6-transduced and E7-transduced cells (although not necessarily to the same extent). For all the spots in this group, z_*i *_was also less than zero (z_*i *_< 0 and significant for circles, z_*i *_< 0 but non-significant for squares). This indicates that although the spots were up-regulated in the E6/7-transduced cells, the effect was not as great as predicted based on the observed x_*i*_, y_*i *_values, assuming independent E6 and E7 effects. Identified proteins in this group are listed in Table 2, and include an oncogene, heat shock proteins, a cytoskeletal protein, a regulator of apoptosis, a translation elongation factor, and other structural proteins and enzymes (see Discussion).

Above and in back of the central grey spots are 88 spots (blue) that were evaluated as significant and share the common property that x_*i *_< 0, y_*i *_< 0, that is, expression was down-regulated in E6- and E7-transduced cells. All spots in this group also shared the property z_*i *_> 0 (z_*i *_significant for circles, non-significant for squares). That is, the effect in E6/7-transduced cells was less than predicted, assuming independent E6 and E7 effects. Identified proteins in this group include a serine protease inhibitor, an apoptotic regulator, a number of intermediate filament proteins associated with differentiated epithelial cells, and several metabolic enzymes (see Discussion).

There were also only 10/741 outliers that were significantly altered in the presence of one oncogene and oppositely regulated in cell cultures expressing the other viral oncogene. These appear as turquoise and green points in the plot. We identified five of these at the molecular level. Four were down-regulated in E6-transduced cells and up-regulated in E7-transduced cells, whereas one was up-regulated in E6-transduced cells and down-regulated in E7-transduced cells. The identities of these proteins and possible reasons for these distinctive patterns of regulation are considered in the Discussion.

It was notable that there were only five outliers for which the increment in expression in E6/7-transduced cells was significantly greater than predicted based on individual effects in E6-transduced and E7-transduced cells (i.e., the "E6/7 interaction term" (z_*i*_,) was significant, *and *x_*i*_, y_*i*_, and z_*i *_all have the same algebraic sign). We identified two of these at the molecular level, and both proved to be charge isoforms of HSPB1 (Table 2).

### Shifts between protein charge isoforms

**Figure 5:**
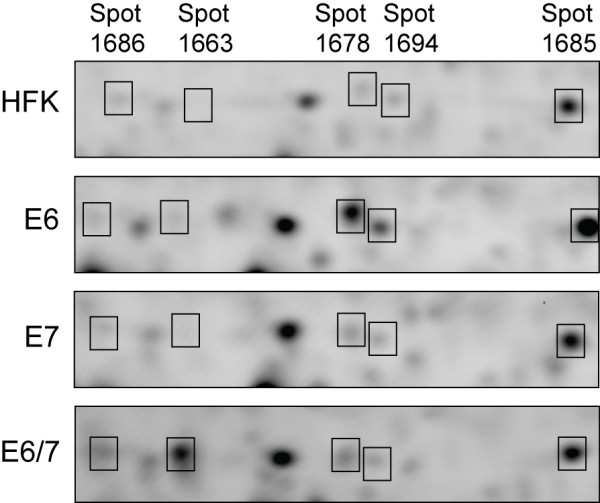
**Enlarged view of the region of the 2D gels containing HSPB1 charge forms**. Representative gels from the HFK, E6, E7, and E6/7 sample groups are shown. Each panel represents one of the sample groups. Images are of the Cy5 (sample) channel only. Boxes are labeled according to master spot number. The gel is oriented as in Figure 2. The region shown spans from approximately pH 5.5 to 6.0.

2D gel analysis provides an ability to detect instances where a treatment leads to a shift in the distribution of charge isoforms for a given protein. Charge isoforms typically manifest as a set of spots that migrate differently in the first dimension but nearly identically in the second. There were two notable instances where nearby, differentially regulated spots were verified as protein charge isoforms by mass spectrometry. Both instances involved stress proteins: HSPA1 and HSPB1 (Table 1). Representative images of the region of the 2D gels containing HSPB1 are shown in Figure 5. Based on its mobility relative to proteins in surrounding areas of the gel, Spot 1685 probably corresponds to unmodified HSPB1, whereas the other four spots have acidic modifications. As noted in the preceding section, two of the HSPB1 spots (1686 and 1663) showed significantly greater expression in E6/7-transduced cells than predicted based on results in individually transduced populations, and this is readily evident from inspection of the gel images. The expression pattern is consistent with (but does not prove) the existence of one charge modification associated with E6 expression, which shifts the unmodified HSPB1 into spots 1678 and 1694, and a second modification associated only with E6/7 co-expression, which shifts this material into spots 1686 and 1663.

### Protein interaction map

**Figure 6:**
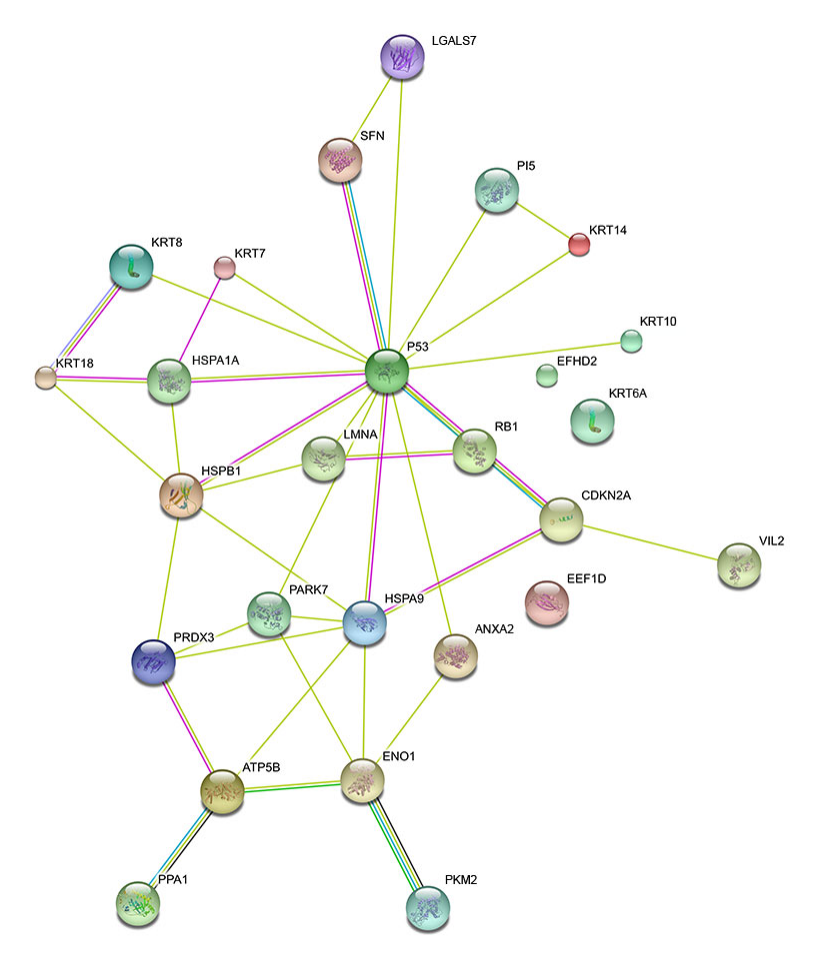
**Protein interaction map**. Map was prepared using the STRING web tool () using default parameters and the accession for identified proteins, human p53, and human Rb proteins. Colored lines denote interactions.

As an additional way to examine the relationship between identified proteins, we used the STRING tool  to prepare an interaction map (Figure 6). We included within the map p53 and Rb proteins, as these are known to be key mediators of E6 and E7 effects, respectively. Of the 24 unique identified proteins, 21 showed connectivity with at least one other protein in the map. In general, proteins with high connectivity are likely to be influential in the operation of biological networks. As might be expected, p53 has the highest connectivity (14 interactions). The molecular chaperones displayed high connectivity not only with p53, but with other identified proteins (e.g., 6 interactions in addition to p53 for HSPA9, 5 for HSPB1). Interestingly, although none of the metabolic and stress response enzymes interact directly with p53 or Rb, they showed high connectivity with other identified proteins (5 interactions for enolase and 4 each for ATP synthase and peroxiredoxin 3).

## Discussion

In this study, we compared protein expression patterns in isogenic populations of primary HFKs and human keratinocytes immortalized by the expression of HPV 16 E6 and E7 oncoproteins. Large-scale proteomic analysis, using 2D-DIGE, provided insight into patterns and trends that would not have been apparent from studies of individual proteins. Results showed that although 170 out of 741 (23%) of the tracked proteomic features differed significantly between oncogene-transduced and primary cells, most of the changes were in the same direction and of comparable magnitude in all three transduced populations (E6, E7, or both). This phenotypic convergence was observed despite the very different mechanisms of action of E6 and E7. We suggest that these changes may not be directly associated with E6 or E7 expression, but rather are characteristic of immortalized epithelial cells, independent of the event originally driving the immortalization process. This interpretation is supported by a literature analysis, which indicates that many of the identified up- and down-regulated spots show similar patterns of alteration in tumors and tumor-derived cell lines that do not express viral oncogenes.

As an example, one of the up-regulated spots was the oncoprotein DJ-1 (PARK7), which transforms mouse 3T3 cells *in vitro *[32] and is also elevated in many non-virally induced human cancers, including lung, breast, ovarian, thyroid, and pancreatic cancers [33-36]. Selection for DJ-1 expression in cancer probably reflects its anti-apoptotic function and interaction with the PTEN signaling pathway [33,34]. There are at least five other examples of up-regulated spots that have previously been described as human epithelial cancer markers: ezrin (VIL2), a cytoskeletal protein linked to metastasis [37,38]; EF-hand domain containing protein-D2 (swiprosin-1), which regulates BCR apoptotic signaling [39]; α-enolase, a glycolytic enzyme that is elevated in cancer cells [40-43]; Elongation factor-1δ, a translation factor and proto-oncogene [44-46]; and Peroxiredoxin-3, which responds to elevated mitochondrial peroxide in cancer cells [47-51].

Among the down-regulated spots were 14-3-3 protein σ, which is a tumor suppressor that is commonly silenced in spontaneous human cancers ([52], reviewed in [53]); Cytokeratins 6 and 14, which have previously been shown to decrease in E6/7-expressing cell cultures and in many epithelial cancers, including cervical cancer [54-57]; and maspin (serpin B5), which is a serine protease inhibitor that has been observed to decrease in an E6/7-expressing *in vitro *model, in the progression of normal cervical epithelium to high-grade intraepithelial lesions and cervical cancer, and in non-virally induced breast cancer [58-60]. Maspin may be selected against because of its ability to influence cell adhesion, motility, angiogenesis, and apoptosis [61]. It is notable that maspin was down-regulated in E7-, as well as E6- and E6/7-transduced cultures. Maspin expression is positively regulated by p53; thus, suppression of maspin in E6- and E6/E7-expressing cultures is consistent with an E6-mediated decrease in p53 expression. The decrease in maspin in E7-expressing cultures is not, however, readily explained by this mechanism, as p53 levels typically increase, rather than decrease, in E7-expressing cells [18,62].

A few proteins in our analysis stand out because they did not follow the common regulatory pattern. Expression of these proteins is evidently directly associated with E6 or E7 expression, rather than indirectly associated with immortalization. The presence of the cyclin-dependent kinase inhibitor p16^INK4a ^in the group of E7-associated genes supports this assumption and can be regarded as an internal control, since p16 is known to be up-regulated as a direct consequence of E7-mediated dissociation of the Rb/E2F/HDAC complex (reviewed in [63]). The observed down-regulation of p16^INK4a ^in association with E6 is unexplained, but is consistent with a prior report [15]. Galectin-7 is another example of a protein that is induced by E7 but suppressed by E6. Galectin-7, which has pro-apoptotic and growth suppressive functions, is dependent on p53 for expression, which may explain its down-regulation in the E6-expressing cells [64,65] and up-regulation in the E7-expressing cells. Galectin-7 is down-regulated in E6/7-transfected cells, consistent with its striking down-regulation in cervical high-grade intraepithelial neoplasia [58] and the known low level of p53 in these tumors. Cytokeratin-7, a protein that is present in cervical cancer but not normal stratified squamous epithelia [66,67], increased in E7-transduced cells, but decreased in E6-transduced cells. A possible explanation is its reported ability, unique among the cytokeratins, to bind and stabilize E7 mRNA[68], which might provide selective pressure for overexpression in E7-transduced cells. Interestingly, Cytokeratin-7 is present in up to 87% of cervical cancers, whereas it is absent from other epithelial cancers [69,70].

There were five significant instances in which cellular protein expression in E6 and E7 co-expressing cells was increased over the sum of effects observed in cells expressing either E6- or E7 alone. Two spots in this category (spots 1663 and 1686) were identified as isoforms of HSPB1. Both migrated as more acidic than expected based on the pI calculated from their primary sequence. The acidic shift could be attributable either to phosphorylation or lysine acetylation, which have both been reported in HSPB1 [71,72]. We failed to detect two peptides, one spanning the Ser 78 and Ser 82 phosphorylation sites, and the other spanning the Ser 15 phosphorylation site, in tryptic digests of spots 1663 and 1686, whereas we readily detected these in digests of the other HSPB1 spots. This suggests, but does not prove, that phosphorylation occurred at Ser 15, Ser78, and/or Ser82. Ser 15, 78, and 82 are known targets of MAPKAPK-2, whereas Ser 82 is a target of the AKT kinase only [73]. It will be of interest to examine the activation state of these kinases further in HPV E6/7-expressing cells.

In parallel clinical proteomic studies, we have noted a complex pattern of HSPB1 regulation. This protein is present at high levels in cornified epithelium, consistent with its function as a cornification chaperone [74]. It declines in high-grade intraepithelial neoplasia and shows a bimodal distribution in invasive cancer, with high levels in some patients, but absent in others [31]. It will be interesting to investigate this phenomenon further and to determine whether the same sites are modified in cancer tissue as are modified in the cell culture model.

## Conclusion

Mucosal squamous cell carcinomas are a leading cause of cancer death, and in many cases are linked to the expression of high-risk type HPV oncogenes. One of the goals of proteomic research is to identify features of squamous cell cancer that are attributable to long-term HPV oncoprotein expression and that might be potential targets for therapeutic intervention. We found that a small fraction of features in the observable proteome were oppositely regulated by E6 or E7 proteins and that an even smaller fraction showed evidence of cooperative regulation. We hypothesize that these proteins are directly regulated by viral oncoproteins and that they may distinguish HPV-driven cancers from cancer in general. We identified p16, Galectin-7, Cytokeratin-7, and HSPA1A as novel members of a set of proteins that are differentially regulated by E6 and E7. The presence of the known E7 target p16^INK4a ^in this set of outliers reinforces its relevance of HPV-dependent gene expression. We also identified post-translationally modified forms of HSPB1 as products of E6 and E7 cooperation.

The incidence of HPV infection is much higher than HPV-driven cancers, i.e., most infections are self-limiting and clear spontaneously. An important goal of proteomics research is to identify features that distinguish cells that are merely virus-infected from those that have undergone initial steps of transformation. Regulators of cell growth and apoptosis identified in the "main sequence" of proteins are candidates to be such features. Based on this criterion, DJ-1, ezrin, Serpin B5, and annexin A2, among others, are of interest as potential markers of progression of HPV-infected cells to HPV-transformed cells.

## Methods

### Cell cultures

Primary human keratinocytes were derived from individual neonatal foreskins and grown in KSF medium (Invitrogen/GIBCO, Carlsbad, CA) supplemented with gentamycin [75]. Cells were infected with amphotrophic LXSN retroviral vectors expressing HPV 16 E6, E7, or both E6 and E7 oncogenes [26,76]. Retrovirus-infected cells were selected in 100 μg/ml G418 for 10 days, then passaged up to twice weekly at 1:5 dilutions. Each passage thus corresponds to approximately 2.3 population doublings. Gene transfer and viral mRNA expression were verified by polymerase chain reaction (PCR) and reverse transcriptase (RT)-PCR.

### 2D-DIGE

To provide biological replicates, viral oncogene-expressing cultures were analyzed separately from two independent pools derived from different passage numbers of the cultures. Lysates were generated from E6-transduced cells at passage numbers 152 and 177, E7-transduced cells at passage numbers 77 and 98, and E6/7-transduced cells at passage numbers 131 and 157. Each lysate was divided into three technical replicates. Each oncogene-expressing culture was therefore represented with six-fold redundancy after data were pooled for analysis.

Cells were trypsinized, and trypsin was inactivated by medium containing 10% fetal bovine serum. Cells were collected by centrifugation, the pellets washed with PBS, and cells were lysed in 7 M urea, 2 M thiourea, 4% CHAPS, 40 mM Tris-HCl pH 8. Aliquots were sonicated on ice and centrifuged for 12000 *g *for 5 min to remove debris. Protein concentrations were determined using a Bradford Assay (Bio-Rad, Hercules, CA). Five μg of protein from each sample was labeled with Cy5 sulfhydryl-reactive dye (0.8 nmol/μg protein, GE Healthcare, Buckinghamshire, UK). For the internal standard, equal amounts of each sample were combined and labeled with Cy3 sulfhydryl-reactive dye (0.8 nmol/μg protein). A 24 cm strip holder containing a pH 3–10 nonlinear IPG strip (GE Healthcare) was used for first dimension electrophoresis. Rehydration of the strip was carried out for 15 h at 20°C with an applied electric field of 30 V, followed by electrophoresis at 500 V for 2 h, 1000 V for 3 h, and 8000 V for 7 h. Strips were equilibrated in 100 mM Tris-HCl (pH 8), 6 M urea, 30% (v/v) glycerol, 2% (w/v) SDS, and 32.5 mM DTT, washed in SDS running buffer, and applied to the top of a 12.5% SDS gel (25 cm × 20 cm × 0.1 cm). Electrophoresis was performed overnight using 2 W per gel. Cy3 and Cy5 images were collected using a GE Healthcare Typhoon 9400 Series Variable Imager.

Quantification and data analysis were performed as described [31]. Comparisons were performed as described in Additional file 1, using Significance Analysis of Microarrays (SAM) to obtain values for parameters representing effects attributable to E6, E7, and biological interactions of E6 and E7. For each comparison, a difference (d) score and a false discovery rate (FDR) were determined by SAM (version 3.0 add-in for Microsoft Excel; available at ) [77]. The d score represents fold change adjusted for a measure of spot-specific variance and a measure of variance within the data set as a whole, while the FDR is based on permuted data sets. Proteins for mass spectrometry analysis were chosen from among the top-ranked proteins in each comparison. Mass spectrometry was performed as described [31].

### Immunoblotting

Cells were suspended in SDS sample buffer and heated for 3 min at 100°C. The protein concentration was determined using a DC Protein Assay kit (Bio-Rad Laboratories, Hercules, CA). Proteins were separated by 14% SDS-PAGE, blotted onto PVDF membranes (Immobilon-P Transfer Membranes, Millipore Corporation, Bedford, MA) and probed with monoclonal antibodies against p16^INK4a ^(G175-405, BD Biosciences, San Jose, CA), Peroxiredoxin-3 (ab16752, Abcam, Cambridge, MA), α-enolase (sc-7455, Santa Cruz Biotechnology, Santa Cruz, CA), or HSPA1(C92F3A-5, Assay Designs Inc., Ann Arbor, MI). Chemiluminescence detection was performed using an ECL kit (Amersham, GE Healthcare). Blots were imaged using the Kodak Image Station CF 440 and analyzed using Kodak 1D 3.5 imaging software (Eastman Kodak, Rochester, NY).

## Competing interests

The authors declare that they have no competing interests.

## Authors' contributions

MAM performed the main proteomic experiments and data analysis and drafted the manuscript. EH assisted in the creation of the cell lines, and HA contributed to the initial phase of the proteomic experiments. RHP created the detailed experimental design and performed statistical analysis. DGF helped conceive the research. WSD supervised data collection and analysis and served as MAM's dissertation advisor. HS is a virologist who conceived the study and created the cell populations that were analyzed. All authors had the opportunity to revise the manuscript for intellectual content and approved the final version.

## Authors' information

MAM performed this work in partial fulfillment of the requirements for the MD/PhD program, Medical College of Georgia. EH was an Assistant Research Scientist and is a molecular biologist. HA was a postdoctoral fellow and is an expert in proteomic analysis. RHP is an Assistant Professor and statistician. DGF is a Professor, family physician, and global health researcher. WSD is a Professor and molecular biologist. HS was an Associate Professor and virologist. WSD is the Principal Investigator for the award that supported the work, and RHP, DGF, and HS were co-investigators.

## Supplementary Material

Additional file 1 Supplemental materials. Click here for file
