# Supplementary material for: Large-scale analysis of protein expression changes in human keratinocytes immortalized by human papilloma virus type 16 E6 and E7 oncogenes
Source: Proteome Sci. 2009 Aug 23;7:29. doi: 10.1186/1477-5956-7-29 (PMC2744660; doi:10.1186/1477-5956-7-29)
Supplement: Additional file 1 — Supplemental materials. [file 1477-5956-7-29-S1.doc]

**Supplemental Material for:**

# Large-scale analysis of protein expression changes in human keratinocytes immortalized by human papilloma virus type 16E6 and E7 oncogenes

Mark A. Merkley^1*^, Ellen Hildebrandt^1†^, Robert H. Podolsky^2^, Hilal Arnouk^1‡^, Daron G. Ferris^3,4^, William S. Dynan^1^, and Hubert Stöppler^1,3§^

^1^Institute of Molecular Medicine and Genetics, Medical College of Georgia, Augusta, GA

^2^Center for Biotechnology and Genomic Medicine, Medical College of Georgia, Augusta, GA

^3^Department of Obstetrics and Gynecology, College of Georgia, Augusta, GA

^4^Department of Family Medicine, Medical College of Georgia, Augusta, GA

^†^Current address: Department of Microbiology and Immunology, Louisiana State University Health Sciences Center, Shreveport, Louisiana 71130.

^‡^Current address: Division of Hematology/Oncology, Department of Medicine, University of Alabama at Birmingham, Birmingham, Alabama 35294

*Submitting author. This author takes responsibility for the article during submission and peer review. Mark A. Merkley, IMMAG CB-3002, Medical College of Georgia, 1120 15th St., Augusta GA 30907. Email: [mmerkley@students.mcg.edu](mailto:mmerkley@students.mcg.edu)

^§^Principal corresponding author:

Hubert Stöppler, UCSF Helen Diller Family Comprehensive Cancer Center, Box 0808, University of California San Francisco, San Francisco, CA 94143-0808. Email: [stopplerh@cc.ucsf.edu](mailto:stopplerh@cc.ucsf.edu)

**DERIVATION OFx*_i_*, y*_i_*, AND z*_i_* PARAMETERS**

**Definitions**

- Let the abbreviations HFKs represent primary human foreskin keratinocytes, E6 represent E6-transduced HFKs, E7 represent E7-transduced HFKs, and E6/7 represent E6/7-transduced HFKs.
- Let **log_2_(*Er_ik_)***represent a mean log_2_-transformed expression value for spot ***i***in experimental group ***k***, relative to corresponding spot ***i*** in the internal standard.
- Let Direct Comparison 1 be the difference between E6 and HFK = **log_2_(*IR_i,E6_*) - (log_2_(*IR_i,HFK_*)**
- Let Direct Comparison 2 be the difference between E7 and HFK = **log_2_(*IR_i,E7_*)- (log_2_(*IR_i,HFK_*)**
- Let Direct Comparison 3 be the difference between E6/7 and HFK = **log_2_(*IR_i,E6/7_*)- (log_2_(*IR_i,HFK_*)**
- Let Grouped Comparison A be the difference between summed expression in groups with E7 and summed expression in groups without E7

= **[(log_2_(*IR_i,E7_*) + log_2_(*IR_i,E6/7_*)] – [(log_2_(*IR_i,HFK_*) + log_2_(*IR_i,E6_*)]**

- Let Grouped Comparison B be the difference between summed expression in groups with E6 and summed expression in groups without E6

= **[(log_2_(*IR_i,E6_*) + log_2_(*IR_i,E6/7_*)] – [(log_2_(*IR_i,HFK_*) + log_2_(*IR_i,E7_*)]**

- Let Grouped Comparison C be the difference of summed expression in groups bearing 0 or 2 oncogenes (i.e. HFK and E6/7) and summed expression in groups bearing 1 oncogene only

= **[log_2_(*IR_i,HFK_*) + log_2_(*IR_i,E6/7_*)] – [(log_2_(*IR_i,E6_*) + log_2_(*IR_i,E7_*)]**

- Let **x*_i_*** represent the log_2_-transformed increments in expression of spot ***i*** associated with the presence of E6. Let ***y_i_***represent the log_2_-transformed increment in expression of spot ***i*** associated with the presence of E7.
- Let ***z_ip_*** represent a log_2_-transformed increment of expression associated with E6/7 co-expression cells (i.e., difference between observed expression in E6/7-transduced cells and predicted expression based on sum of increments associated with each oncogene individually).

**Determination of values for each parameter based on direct and grouped comparisons.**

We can write:

***Eqn 1:* log_2_(*IR_i,E6_) =* log_2_(*IR_i,HFK_)***+ ***x_i_***

***Eqn 2:* log_2_(*IR_i,E7_) =* log_2_(*IR_i,HFK_)***+ ***y_i_***

***Eqn 3:***  **log_2_(*IR_i,E6/7_) =* log_2_(*IR_i,HFK_)*** + ***x_i_*** + ***y_i_*** + **z*_i_***

Substituting equations **1-3** we obtain the following:

Direct comparison 1 = ***x_i_***

Direct comparison 2 = ***y_i_***

Direct comparison 3 = ***x_i_*** + ***y_i_*** + **z*_i_***

Grouped Comparison A= 2 ***y_i_*** + **z*_i_***

Grouped Comparison B= 2 ***x_i_*** + **z*_i_***

Grouped Comparison C= **z*_i_***

Direct Comparison 1, Direct Comparison 2, and Grouped Comparison C lead directly to values for parameters of biological interest – ***x_i_***,***y_i_***, and**z*_i_***, corresponding to the effects of E6, E7, and E6/7 interaction respectively. Grouped comparisons A and B provide alternative ways to derive values for ***x_i_*** and ***y_i_*** when **z*_i_***≈0. Grouped comparisons have greater statistical power (the entire data set is used) and are therefore preferred over direct comparison when **z*_i_***≈0.

**Evaluation of statistical and biological significance.** Significance was assessed using the decision tree in Supplemental Figure 1. Statistical significance was determined using SAM,with a FDR threshold of 20% for **z*_i_*** (Grouped Comparison C) and 5% for **x***_i_*and **y*_i_*** (Grouped Comparisons A and B). The rationale for using different FDR thresholds is that significance of **z*_i_***is used primarily to decide which further tests are appropriate, whereas significance of **x*_i_***and **y*_i_***is used directly as a criterion for identification of spots of interest.


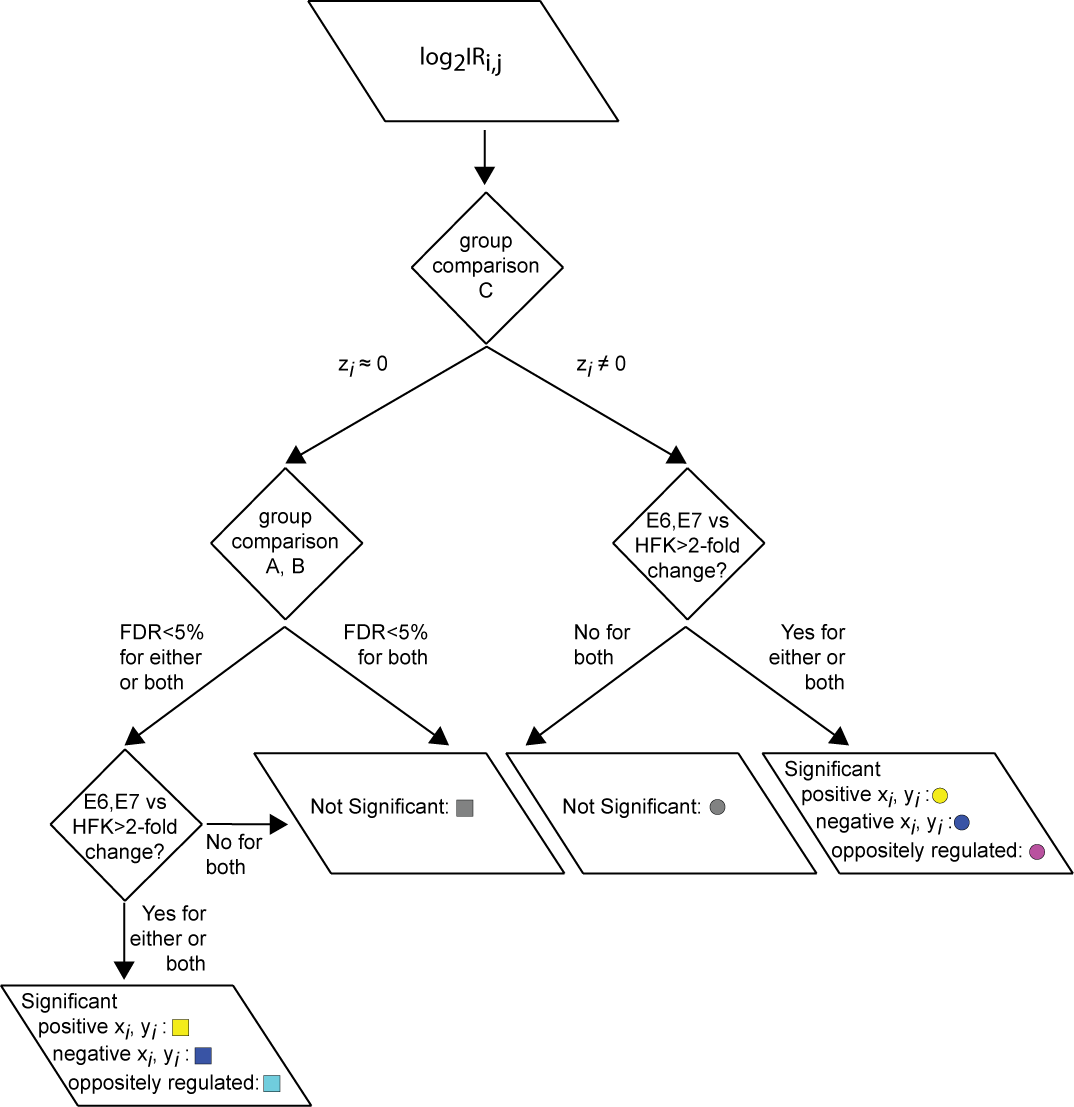


**Supplemental Figure 1.** Decision tree for significance analysis of proteins in data set.

**Examples shown in supplemental Figure 2 are as follows:**


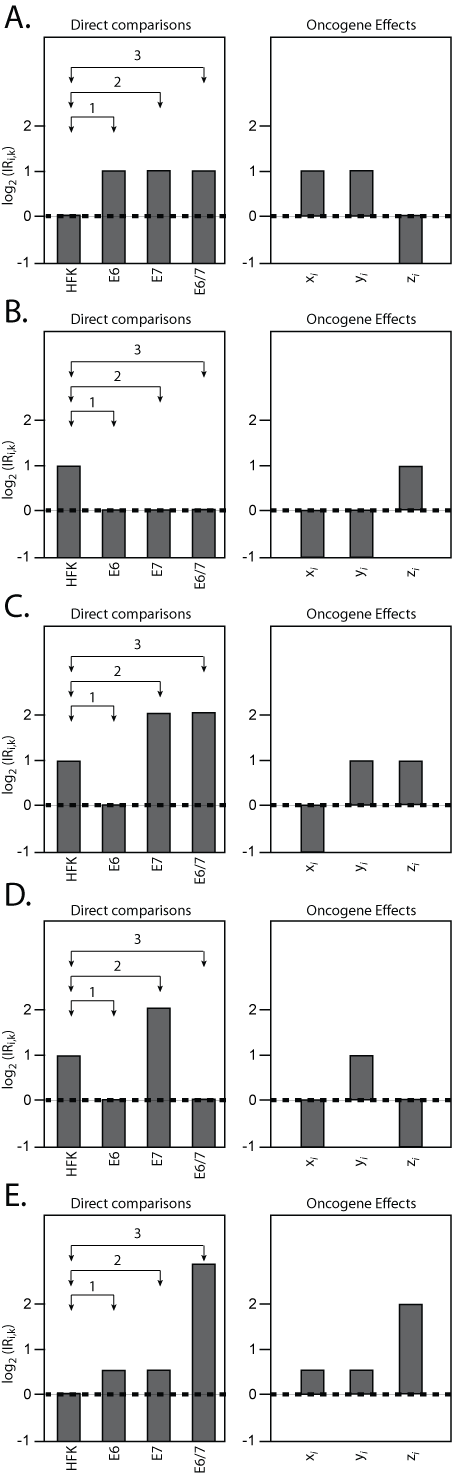


**Supplemental Figure 2.** Expression parameters obtained in hypothetical illustrations.

1. Expression is twice as high in all 3 oncogene-transduced populations, as compared to HFK. In this case, **x*_i_***=**y*_i_***,=1 (2^1^= 2-fold increase). The predicted effect in E6/7-transduced cells, assuming independent mechanisms of action, is 2^1+1^=4-fold. However, the actual effect is only 2-fold. Thus, the sign of **z*_i_***is opposite to **x*_i_*** and **y*_i_***, (combined effect less than predicted assuming independent mechanisms of action).
2. Similar to A, except that the expression is down-regulated in all 3 oncogene-transduced populations. **x*_i_***=**y*_i_***,=-1 (2^-1^= 2-fold decrease). The predicted effect in E6/7-transduced cells, assuming independent mechanisms of action, is 2^(-1-1)^=4-fold decrease. However, the actual decrease is only 2-fold. Thus, the sign of **z*_i_***is opposite to **x*_i_*** and **y*_i_***, (combined effect less than predicted assuming independent mechanisms of action).
3. Expression is half as great in HFKs when E6-transduced , twice as great when E7-transduced , and twice as great when E6/7-transduced . **x*_i_*** =-1, **y*_i_*** =1. The predicted effect in E6/7 cells is 2^(-1+1)^=2^0^=1 (no effect). The actual change in E6/7 cells is a 2-fold increase, so **z*_i_*** =1.
4. Similar to C, except that expression, except that E6/7 cells have half as much expression as HFK. Again, **x*_i_*** =-1, **y*_i_*** =1, predicted effect in E6/7 is no change. Actual effect is 2-fold decrease, so **z*_i_*** =-1.
5. Expression is slightly elevated in E6-transduced and E7-transduced (2^0.5^=1.4-fold increase) and strongly elevated in E6/7-transduced (2^3^= 8 fold increase)exceeding the sum of the spot intensities in E6 and E7-expressing cultures (**z*_i_*** =2). (Examples (A) and (B) correspond to a pattern of regulation that was very common in the experimental data set. Examples (C) and (D) are similar to outliers, such as p16^ink4a^ and Galectin 7. Example (E) was very rare in the experimental dataset: only 5/741 spots demonstrated signal intensities in E6/7-expressing cultures thatsignificantly exceeded the sum of the spot intensities in E6 and E7-expressing cultures.

**Supplemental Table 1: protein identification**

| **spot** | **Protein Name** | **Accession Number** |  | **Molecular Weight** | **Calculated pI** | **Peptides Identified** | **Percent coverage** |
| --- | --- | --- | --- | --- | --- | --- | --- |
| 266 | Ezrin | P15311 | VIL2 | 69199 | 5.94 | 25 | 45 |
| 366 | Heat shock 70 kDa protein 1 | P08107 | HSPA1A | 70009 | 5.48 | 14 | 27 |
| 377 | Heat shock 70 kDa protein 1 | P08107 | HSPA1A | 70009 | 5.48 | 19 | 36 |
| 382 | Stress-70 protein, mitochondrial | P38647 | HSPA9 | 73682 | 6.03 | 11 | 22 |
| 439 | Progerin (Lamin A/C) | Q6UYC3 | LMNA | 69207 | 6.22 | 18 | 31 |
| 534 | Keratin, type II cytoskeletal 6D | P02538 | KRT6A | 42442 | 5.29 | 10 | 27 |
| 545 | Pyruvate kinase isozymeM2 | P14618 | PKM2 | 57769 | 7.95 | 12 | 30 |
| 560 | Keratin, type II cytoskeletal 6D | P02538 | KRT6C | 42442 | 5.29 | 15 | 40 |
| 572 | Keratin, type II cytoskeletal 6D | P02539 | KRT6C | 42442 | 5.29 | 14 | 39 |
| 645 | Keratin, type II cytoskeletal 7 | P08729 | KRT7 | 51255 | 5.5 | 17 | 42 |
| 646 | Keratin, type II cytoskeletal 7 | P08729 | KRT7 | 51255 | 5.5 | 15 | 34 |
| 672 | Keratin, type I cytoskeletal 14 | P02533 | KRT14 | 51458 | 5.09 | 16 | 42 |
| 739 | Keratin, type II cytoskeletal 8 | P05787 | KRT8 | 41083 | 4.94 | 17 | 54 |
| 766 | α-enolase | P06733 | ENO1 | 47008 | 6.99 | 7 | 18 |
| 775 | ATP synthase subunit b, mitochondrial | P006576 | ATP5B | 56525 | 5.26 | 19 | 48 |
| 777 | α-enolase | P06733 | ENO1 | 47008 | 6.99 | 7 | 24 |
| 778 | α-enolase | P06733 | ENO1 | 47008 | 6.99 | 10 | 34 |
| 781 | α-enolase | P06733 | ENO1 | 47008 | 6.99 | 8 | 27 |
| 903 | Serpin B5 (maspin) | P36952 | PI5 | 42111 | 5.72 | 11 | 39 |
| 915 | Keratin, type I cytoskeletal 18 | P05783 | KRT18 | 47305 | 5.27 | 16 | 44 |
| 1110 | Annexin A2 | Q8TBV2 | ANXA2 | 38449 | 7.56 | 12 | 44 |
| 1111 | Annexin A2 | Q8TBV2 | ANXA2 | 38449 | 7.56 | 13 | 45 |
| 1121 | Annexin A2 | Q8TBV2 | ANXA2 | 38449 | 7.56 | 11 | 30 |
| 1186 | Inorganic pyrophosphatase | Q15181 | PPA1 | 32639 | 5.54 | 12 | 57 |
| 1451 | EF-hand domain-containing protein D2 | Q96C19 | EFHD2 | 26680 | 5.15 | 9 | 35 |
| 1586 | 14-3-3 protein σ | P31947 | SFN | 27757 | 4.68 | 9 | 39 |
| 1663 | Heat shock protein B1 | P04792 | HSPB1 | 22768 | 5.98 | 8 | 41 |
| 1678 | Heat shock protein B1 | P04792 | HSPB1 | 22768 | 5.98 | 9 | 44 |
| 1685 | Heat shock protein B1 | P04792 | HSPB1 | 22768 | 5.98 | 9 | 44 |
| 1686 | Heat shock protein B1 | P04792 | HSPB1 | 22768 | 5.98 | 7 | 36 |
| 1694 | Heat shock protein B1 | P04792 | HSPB1 | 22768 | 5.98 | 9 | 44 |
| 1721 | Keratin, type I cytoskeletal 10 | P13645 | KRT10 | 58792 | 5.09 | 8 | 15 |
| 1839 | Thioredoxin-dependent peroxide reductase, mitochondrial | P30048 | PRDX3 | 27607 | 7.67 | 5 | 25 |
| 1849 | Protein DJ-1 | Q99497 | PARK7 | 19834 | 6.33 | 5 | 37 |
| 1859 | Protein DJ-1 | Q99497 | PARK7 | 19834 | 6.33 | 7 | 38 |
| 2402 | Cyclin-dependent kinase inhibitor 2A, isoforms 1/2/3 (p16) | P42771 | CDKN2A | 16533 | 5.52 | 4 | 31 |
| 2597 | Galectin-7 | P47929 | LGALS7 | 14635 | 7.52 | 6 | 59 |
| 2967 | Keratin, type II cytoskeletal 6D | P02538 | KRT6A | 42442 | 5.29 | 9 | 25 |
| 2983 | Elongation factor 1-δ | P29692 | EEF1D | 30972 | 4.9 | 9 | 45 |
